# An omentum-inspired 3D PEG hydrogel for identifying ECM-drivers of drug resistant ovarian cancer

**DOI:** 10.1063/1.5091713

**Published:** 2019-06-26

**Authors:** Elizabeth A. Brooks, Maria F. Gencoglu, Daniel C. Corbett, Kelly R. Stevens, Shelly R. Peyton

**Affiliations:** 1 Department of Chemical Engineering, University of Massachusetts Amherst, N540 Life Science Laboratories, 240 Thatcher Road, Amherst, Massachusetts 01003-9364, USA; 2 Department of Bioengineering, University of Washington, Box 355061, Seattle, Washington 98195-5061, USA

## Abstract

Ovarian cancer (OvCa) is a challenging disease to treat due to poor screening techniques and late diagnosis. There is an urgent need for additional therapy options, as patients recur in 70% of cases. The limited availability of clinical treatment options could be a result of poor predictions in early stage drug screens on standard tissue culture polystyrene (TCPS). TCPS does not capture the mechanical and biochemical cues that cells experience *in vivo*, which can impact how cells will respond to a drug. Therefore, an *in vitro* model that captures some of the microenvironment features that the cells experience *in vivo* could provide better insights into drug responses. In this study, we formed 3D multicellular tumor spheroids (MCTS) in microwells and encapsulated them in 3D omentum-inspired hydrogels. SKOV-3 MCTS were resistant to Paclitaxel in our 3D hydrogels compared to a monolayer on TCPS. Toward clinical application, we tested cells from patients [ovarian carcinoma ascites spheroids (OCAS)] who had been treated with Paclitaxel, and drug responses predicted by using the 3D omentum-inspired hydrogels demonstrated the lack of the Paclitaxel response of these samples. Additionally, we observed the presence of collagen production around the encapsulated SKOV-3 MCTS, but not significantly on TCPS. Our results demonstrated that our 3D omentum-inspired hydrogel is an improved *in vitro* drug testing platform to study the OvCa drug response for patient-derived cells and helped us identify collagen 3 as a potential driver of Paclitaxel resistance in 3D.

## INTRODUCTION

Ovarian cancer (OvCa) is the fifth deadliest cancer for women, and the deadliest gynecological disease for women overall, resulting in an estimated 14 070 deaths in the United States in 2018.^1^ The high mortality rate of OvCa is due to late detection, inadequate screening techniques, and a lack of effective second line therapies.^2^ OvCa is typically detected very late, partially because the disease is asymptomatic until Stage III,^3^ when the cancer cells are no longer confined to the ovaries. At the time of staging laparectomy, metastases that have spread through the peritoneum are present in 70% of patients.^3^ It has been accepted in the field that tumor cells spread into the peritoneal fluid, where they form ovarian carcinoma ascites spheroids (OCAS), and can attach onto the abdominal peritoneum or omentum.^4,5^ While aggregation has been accepted for model OCAS formation, recent work demonstrated that detachment of clusters from the primary tumor may be the more likely source of OCAS in the abdomen.^6^ Regardless of the OCAS formation method *in vivo*, prognosis for OvCa patients has improved little in the last three decades since the introduction of platinum-based treatments.^7^ Although first line therapy results in complete remission in 40%–60% of patients, over 70% of those patients relapse within two years, and there are no effective second line treatments.^8,9^ Additionally, to our knowledge, only four targeted drugs have been approved by the Food and Drug Administration (FDA) in the last ten years for OvCa treatment. There is a clear need for innovation in drug discovery to develop additional OvCa treatments to improve patient outcomes.

A possible reason for the inadequate success in OvCa drug discovery is that current preclinical *in vitro* screening methods are not good predictors of clinical success of drug candidates. Many hypothesize that this is largely because *in vitro* screening uses cell lines grown on tissue culture polystyrene (TCPS).^10,11^ The TCPS surface has no resemblance to the *in vivo* microenvironment chemically, physically, or topologically.^12^ Cancer cells grown in this environment have different morphologies, phenotypes, cellular signaling, and drug responses from cells found *in vivo*.^13^ Three-dimensional (3D) culture models are increasingly used in basic science applications because they offer environments that better resemble *in vivo* conditions,^14,15^ and they can be tuned to model different tissues.^16,17^ Most 3D models consist of hydrogels or similar biomaterials that have a 3D structure inside which cancer cells can be grown.^18–21^ These materials can be functionalized with peptides to mimic cell-extracellular matrix (ECM) interactions, ECM degradability, and other *in vivo* properties.^22,23^

There are several types of 3D models in which cancer multicellular tumor spheroids (MCTS, which resembles OCAS) can be grown *in vitro*.^24^ Incorporating MCTS grown from established cell lines may also better model the *in vivo* microenvironment for drug screening than TCPS. However, the use of established cell lines does not capture disease heterogeneity across patients^25^ and the lack of clinical relevance of cell lines is becoming increasingly apparent^26^ despite their widespread use for *in vitro* studies.^27^ For this reason, *in vitro* models of OvCa tissue coupled with patient-derived OCAS compared with traditional screening methods could improve OvCa drug discovery.^28^ Thus, they would promise fewer false leads and so better drug discovery. While genetic mechanisms of OvCa have been studied,^29^ these findings alone have not been sufficient for explaining the drug response.^30^ The use of primary cells isolated from patients would improve testing of drugs in a more personalized way.^31^ Ascites fluid in the peritoneal cavity of OvCa patients is rich in single malignant cells and OCAS,^32,33^ and this fluid is often drained to relieve the pain it causes. In this study, we evaluated the response of SKOV-3 OvCa MCTS and patient-derived OCAS to several drugs across different mechanisms of action in synthetic, tailorable hydrogels. Through this study, we revealed a nonintuitive response of patient cells to these drugs, and we nominate collagen 3 as a particularly interesting ECM protein potentially driving resistance to Paclitaxel in 3D. These hydrogels are easy to create and support the viability and survival of patient-derived OCAS, and we propose this as a useful system to preclinically screen drug candidates.

## RESULTS

### OvCa MCTS were viable and proliferative in 3D poly(ethylene glycol)-maleimide (PEG-MAL) hydrogels

**FIG. 1.**
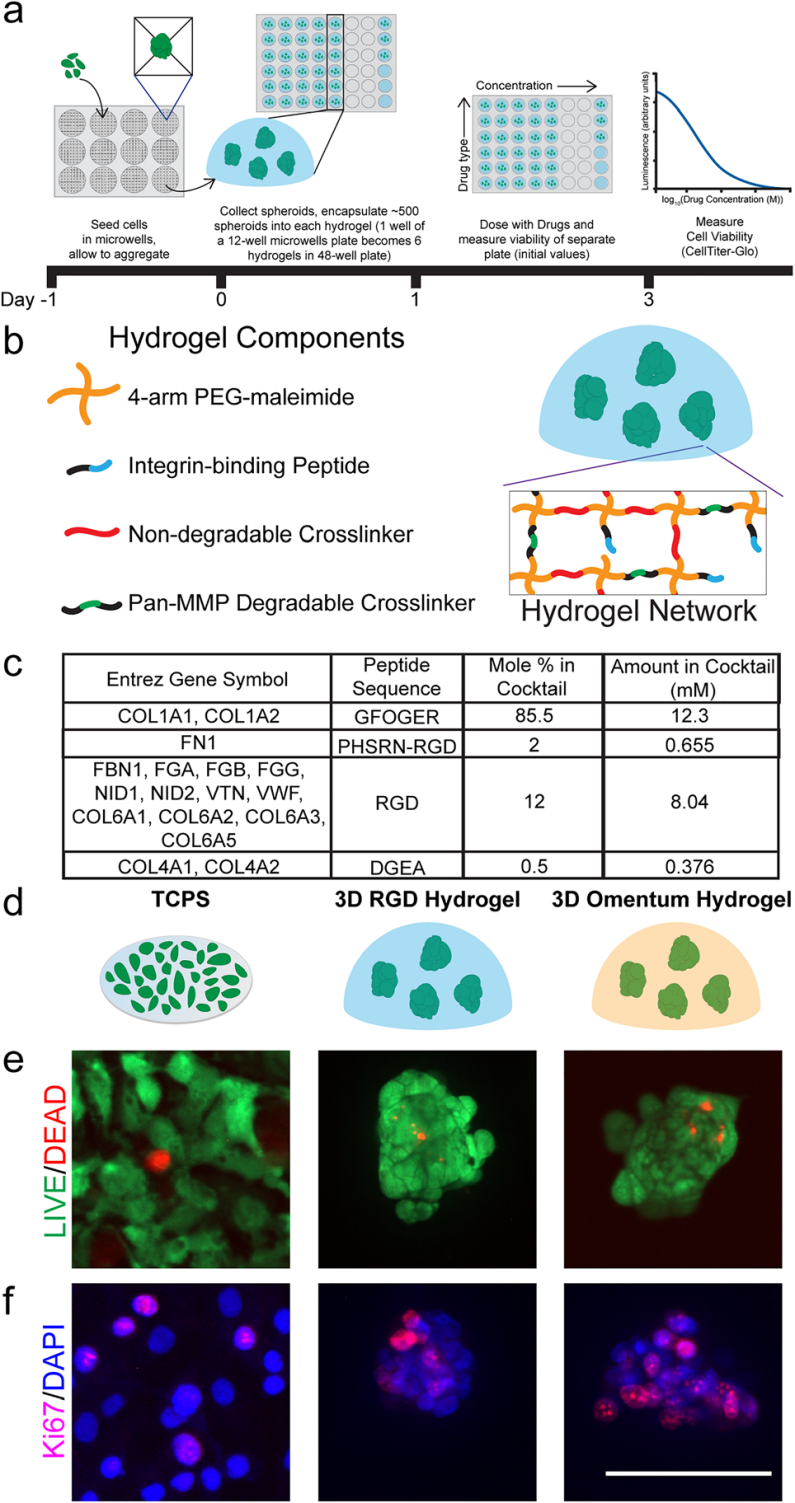
Three biomaterial platforms that were used to evaluate the SKOV-3 drug response. (a) Schematic of the MCTS formation method and the experimental setup for drug screening MCTS encapsulated in 3D PEG-MAL hydrogels. (b) Schematic of the components of a 3D PEG-MAL hydrogel with encapsulated SKOV-3 MCTS within a 3D hydrogel. (c) Omentum-inspired integrin-binding peptide cocktail based on healthy omentum proteomic data. (d) Schematic of cells grown on plastic or encapsulated in a 3D hydrogel model. (e) LIVE (green)/DEAD (red) imaging of cells in each platform one day postencapsulation. (f) Ki67 (pink)/DAPI (blue) staining of cells one day postencapsulation to evaluate cell proliferation. Scale bar = 100 *μ*m. Images are representative from N ≥ 2 independent experiments.

OvCa metastasizes to several sites within the peritoneal cavity.^4^ Here, we chose to focus on the omentum due to the availability of published data on this tissue of OvCa metastasis, which we used to design our 3D hydrogel model. Since OvCa metastasizes to the omentum as aggregated cells,^33^ we hypothesized that a 3D hydrogel with integrin-binding peptides found in the omentum would support the viability and proliferation of OvCa MCTS (Fig. 1). We previously used three methods for creating MCTS from established cell lines,^24^ and here, we chose to use the microwell formation method [Fig. 1(a)]. At the time of the design of this study, aggregation was accepted to be more akin to how OvCa form OCAS (aggregated single cells rather than clonal growth from a single cell) *in vivo* during metastasis,^33^ but aggregates may actually detach from the primary tumor as clusters.^6^ Our previous work demonstrated that the microwell formation method is fast (MCTS form within one day) and resulted in the most uniform size distribution of MCTS.^24^ For this study, we selected the widely used SKOV-3 OvCa cell line to form MCTS. We then chose to encapsulate these MCTS in 3D poly (ethylene glycol)-maleimide (PEG-MAL) hydrogels to capture a physiologically relevant stiffness (approximately 2 kPa)^22,34^ with a high water content, as well as tailored integrin-binding and matrix degradability. Since PEG hydrogels are not inherently degradable by cells, it was necessary to incorporate a cell-degradable cross-linking sequence during hydrogel synthesis to facilitate cell survival and spreading within the matrix. For this study, we selected a Pan-matrix metalloproteinase (Pan-MMP) degradable peptide cross-linker that can be degraded by many MMPs at the glycine-isoleucine bond.^35^ To optimize MCTS survival, we varied the amount of Pan-MMP degradable cross-linking within the hydrogel that contained RGD peptide integrin-binding [Fig. S1(a)]. The total cross-linking was kept at a 1:1 molar ratio of maleimide-functional end groups to thiol-functional end groups by completing the cross-linking solution with linear PEG-dithiol (PDT), while the amount of Pan-MMP degradable cross-linking was varied. From our data, we concluded that 12 mol. % Pan-MMP degradable cross-linking supported MCTS survival and we used this condition for all subsequent experiments unless otherwise noted.

We tested encapsulating MCTS from one (5500 MCTS/ml approximate), two (11 000 MCTS/ml), or three (16 500 MCTS/ml) wells of the 12-well plate containing microwells into six PEG-MAL hydrogels with RGD peptide integrin-binding and 25 mol. % Pan-MMP degradable cross-linking with the remainder of the cross-linking with linear PDT. We found that the ratio of one well from the 12-well plate to six PEG-MAL hydrogels (approximately 500 MCTS per hydrogel) was ideal for a CellTiter-Glo output signal where we would be able to detect changes in the MCTS response with varying drug concentrations. Additionally, we found that the relative change in the output signal from day one to day three was approximately the same for each ratio of wells from the microwells plate to six PEG-MAL hydrogels [Fig. S1(b)]. We used approximately 500 MCTS per PEG-MAL hydrogel for the remainder of this study. This allowed us to test as many conditions as possible using one 12-well microwells plate and did not reduce the quality of the final assay measurement.

We measured the drug response of SKOV-3 MCTS in a 3D PEG-MAL hydrogel with RGD integrin-binding, but we hypothesized that adding tissue specificity could provide a better environment for MCTS survival, and therefore, the drug responses would be more representative of what would occur *in vivo*. We therefore increased the complexity of the 3D hydrogel model by adding additional integrin-binding peptides specific to the omentum, which is the site of many OvCa metastases.^4^ We aimed to create a 3D hydrogel with some tissue specificity without being overly complex, which would potentially limit future applications in drug screening or in a clinical setting. To find the most abundant integrin-binding proteins in the omentum, we mined published mass spectrometry data of healthy omentum tissue.^36^ The full integrin-binding peptide cocktail is described in Fig. 1(c). GFOGER, which is the binding sequence in collagen 1 and other fibrillar collagens,^37^ comprises a majority of the hydrogel, but this cocktail contains RGD, as well as PHSRN-RGD and DGEA representative of other proteins described in Fig. 1(c). For simplicity, we kept the Pan-MMP degradable cross-linker used with the RGD only integrin-binding hydrogel to allow for MCTS to degrade the surrounding hydrogel.

Since this omentum-inspired hydrogel is new to the field, we first ensured that cells were viable and proliferative before drug screening, alongside two other platforms as internal controls [Fig. 1(d)]. As expected, SKOV-3 cells cultured on TCPS showed high viability and proliferation indicated by LIVE/DEAD and Ki67 staining, respectively [Figs. 1(e) and 1(f)]. Both our PEG-MAL hydrogel models supported cell survival with very few dead cells inside the MCTS. Additionally, we observed proliferating cells throughout the MCTS by Ki67 staining [Fig. 1(f)]. These results allowed for subsequent drug screening experiments in these platforms.

### SKOV-3 OvCa MCTS were resistant to Paclitaxel and sensitized to other drugs in the 3D PEG-MAL hydrogels relative to TCPS

**FIG. 2.**
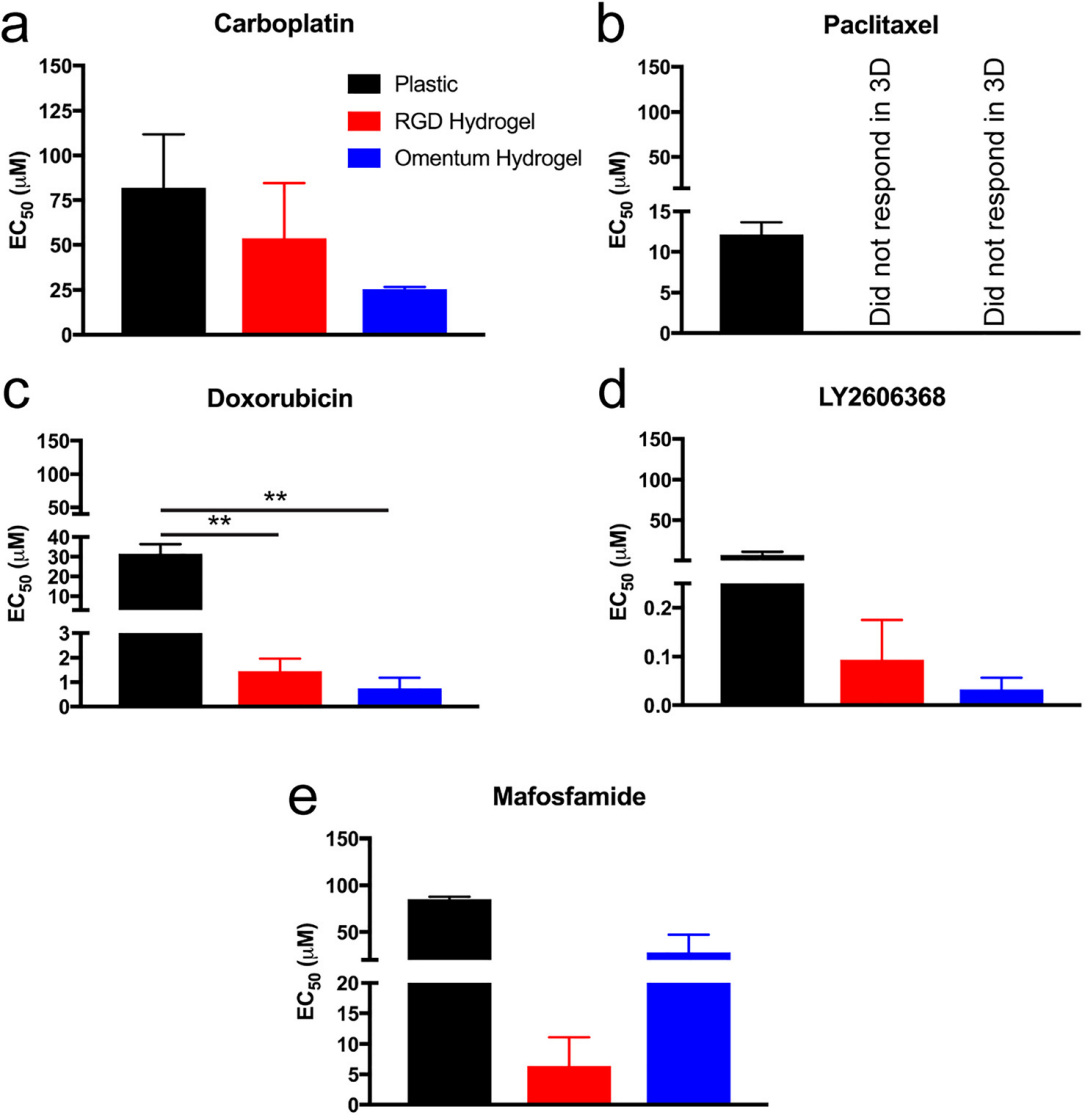
Quantified SKOV-3 drug response reported as EC_50_ on TCPS or as MCTS encapsulated in RGD or omentum-inspired peptide-binding 3D PEG-MAL hydrogels. (a) Carboplatin. (b) Paclitaxel. (c) Doxorubicin. (d) LY2606368. (e) Mafosfamide. Data are reported as the mean ± SEM. The EC_50_ values were calculated using the online GR calculator and from N ≥ 2 independent experiments with N = 3 technical replicates on TCPS and N ≥ 2 technical replicates in hydrogels. Statistical significance where p ≤ 0.01 is denoted with **.

As a control, we first established drug responses of the SKOV-3 cells to five drugs, spanning different mechanisms of action, on TCPS (Figs. 2 and S2-3). We quantified the IC_50_, EC_50_, and GR_50_ (Ref. 38) (Fig. S2) for each cell line and drug combination using the online GR calculator.^39^ We calculated and reported these three parameters because they each provided different information about the potency of the drug on the cell line.^38^ In all cases, the GR_50_ was lower than the IC_50_ and EC_50_ metrics (Fig. S2), because the GR_50_ is quantifying the reduction in the growth rate and not overall inhibition of the cell number. We found that SKOV-3 MCTS did not respond to Paclitaxel in the 3D hydrogels [EC_50_, Figs. 2(b) and S4-5] but had an average EC_50_ of 12.1 *μ*M on TCPS. We chose to report and compare the EC_50_ for each condition since it is the concentration that corresponds to achieve half of the maximal response and does not require at least 50% cell death as the IC_50_ does.^38^ This means that the drug responses across multiple conditions could still be compared if the cells did not grow exponentially over the course of the experiment or if greater than 50% cell death relative to the control was not achieved, which is required for calculating the GR_50_ and IC_50_, respectively. We found that it was not possible to calculate the GR_50_ in many of the 3D cases that we tested. Since Paclitaxel is a first-line therapy for OvCa patients, we were surprised to see a lack of response of SKOV-3 MCTS in our hydrogel models to this chemotherapy. However, OvCa Paclitaxel resistance has been previously reported in another 3D model by Loessner *et al.*^40^ This observation is important because the drug response curve for SKOV-3 with Paclitaxel on TCPS is typical [Fig. S3(b)], making it appear efficacious, but the curve looks strikingly dissimilar in a 3D culture model.

Surprisingly, we found that the MCTS in the hydrogels were more sensitive to all the other drugs that we tested, relative to TCPS (EC_50_, Figs. 2 and S4-5). SKOV-3 MCTS were more sensitive (lower EC_50_) to Carboplatin and Doxorubicin [Figs. 2(a) and 2(c)] in the omentum hydrogel than in the RGD hydrogel. On the other hand, MCTS were more sensitive to LY2606368 and Mafosfamide [Figs. 2(d) and 2(e)] in the omentum hydrogel than on TCPS although MCTS were less sensitive to Mafosfamide in the omentum hydrogel compared to the 3D RGD hydrogel; note that these compounds have not yet become clinically approved for OvCa treatment. An expanded future study with these models and other drugs, both clinically approved and in development, could possibly be used to make correlations between preclinical studies and clinical efficacy in patients.

### Patient-derived OCAS drug sensitivity reflected the real clinical response in omentum-mimicking hydrogels

**FIG. 3.**
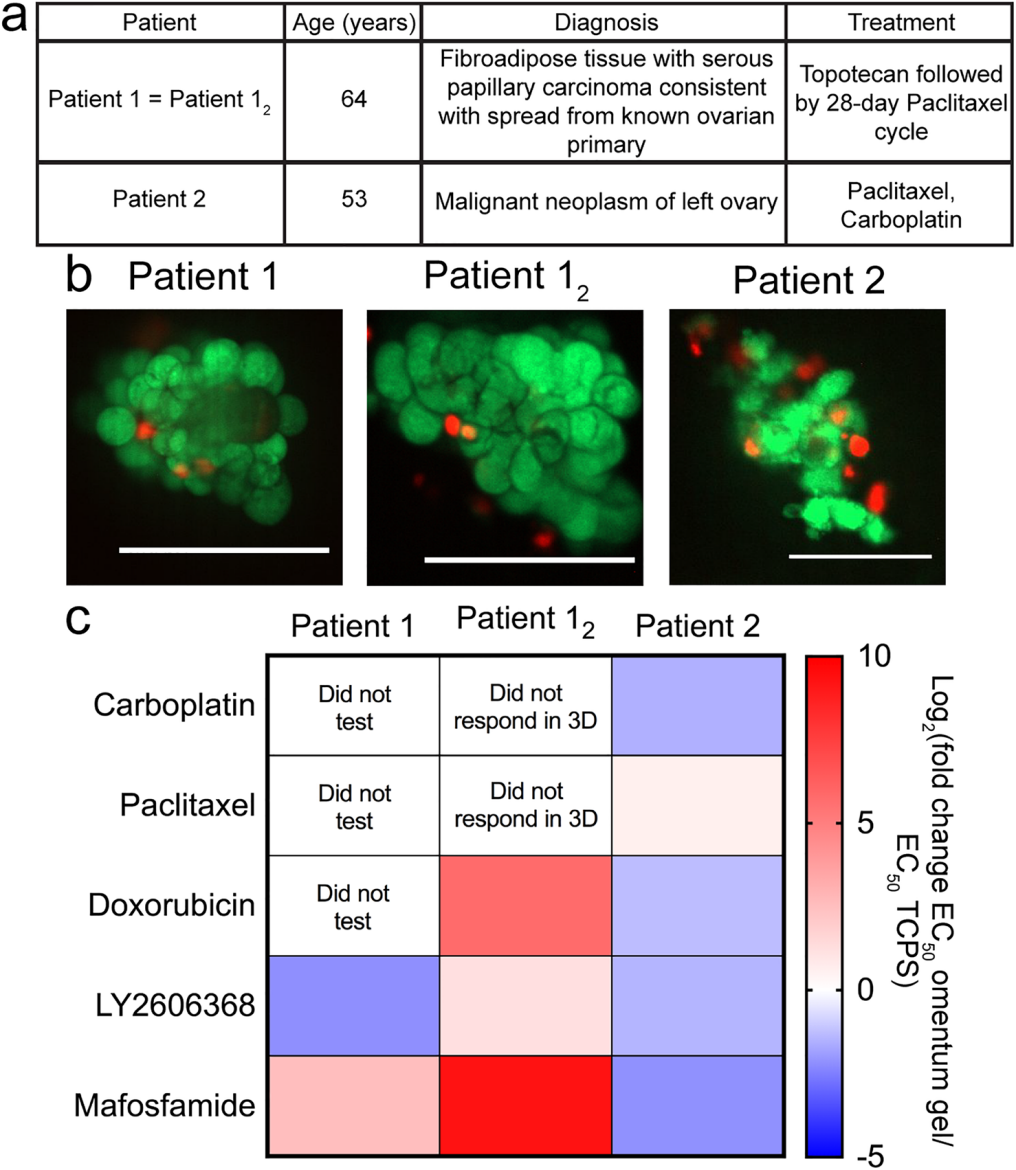
OvCa samples derived from patient ascites were encapsulated in 3D omentum-inspired PEG-MAL hydrogels for drug screening. (a) Pathology report of patient samples. (b) LIVE (green)/DEAD (red) imaging of OCAS in 3D omentum PEGMAL hydrogels one day postencapsulation. Scale bars = 100 *μ*m. Images are representative from the sample. (c) Patient sample drug responses displayed as the log_2_ fold change in EC_50_ in the omentum hydrogel relative to EC_50_ on TCPS. Red and blue indicate the higher and lower EC_50_ values in the 3D omentum hydrogel relative to the EC_50_ on TCPS, respectively.

Our goal was to evaluate how OCAS from patient samples responded to the drugs in our study in the 3D omentum-inspired hydrogel relative to TCPS. We collaborated with the team at UMass Medical School to obtain ascites fluid from OvCa patients who had undergone paracentesis. Ascites is a fluid rich in single cells, OCAS, and soluble factors, which can be processed to collect the different components.^32^ The cells in ascites are a particularly valuable material for drug screening since they are a fairly accessible source of primary cells to study OvCa. To isolate cells and OCAS for drug screening purposes, we processed the ascites using centrifugation, removal of red blood cells, and washing steps, which has been described in detail previously.^24^ We then encapsulated OCAS into 3D omentum hydrogels and compared their 3D drug responses with those of TCPS (Fig. 3). The basic pathology reports for the patient samples are listed in Fig. 3(a). Prior to drug screening, samples were assessed for viability via LIVE/DEAD staining [Fig. 3(b)], which showed high viability across all samples in the 3D PEG-MAL omentum hydrogels. We observed that samples from patient 1 were resistant to Mafosfamide, but sensitive to LY2606368 in the 3D hydrogel relative to TCPS [Figs. 3(c) and S6]. LY2606368 is an inhibitor that is currently in Phase II clinical trials for OvCa.^41,42^ However, upon receiving the next sample (patient 1_2_ was the same as patient 1 with six weeks between sample collection), resistance in 3D vs on TCPS increased for these two drugs. Patient 2 received both Carboplatin and Paclitaxel [Fig. 3(a)]. Our 3D omentum hydrogel model demonstrated sensitivity to Carboplatin, Doxorubicin, LY2606368, and Mafosfamide relative to TCPS and only slight resistance to Paclitaxel. If patient 2 did not respond to these drugs in the clinic (Carboplatin and Paclitaxel) and the response was not dependent on geometry, we would expect a lack of responses both on TCPS and in 3D. Because of IRB restrictions, we did not obtain final patient outcome information, but we did see responses to these drugs in our study (Fig. S6). Interestingly, Doxorubicin, LY2606368, and Mafosfamide all appeared to be more effective in 3D vs 2D for the patient 2 sample. Since we observed a decreased response to LY2606368 and Mafosfamide in 3D relative to TCPS with the samples from patient 1, it may indicate that our hydrogel model could be a way to predict potential drug responses of patients as the disease progresses. Screening to obtain this type of information could be a way to help guide future clinical treatment decisions. However, many more samples would be required to establish generalized observations of eventual prognostic value.

### SKOV-3 MCTS expressed ECM proteins in 3D hydrogels

**FIG. 4.**
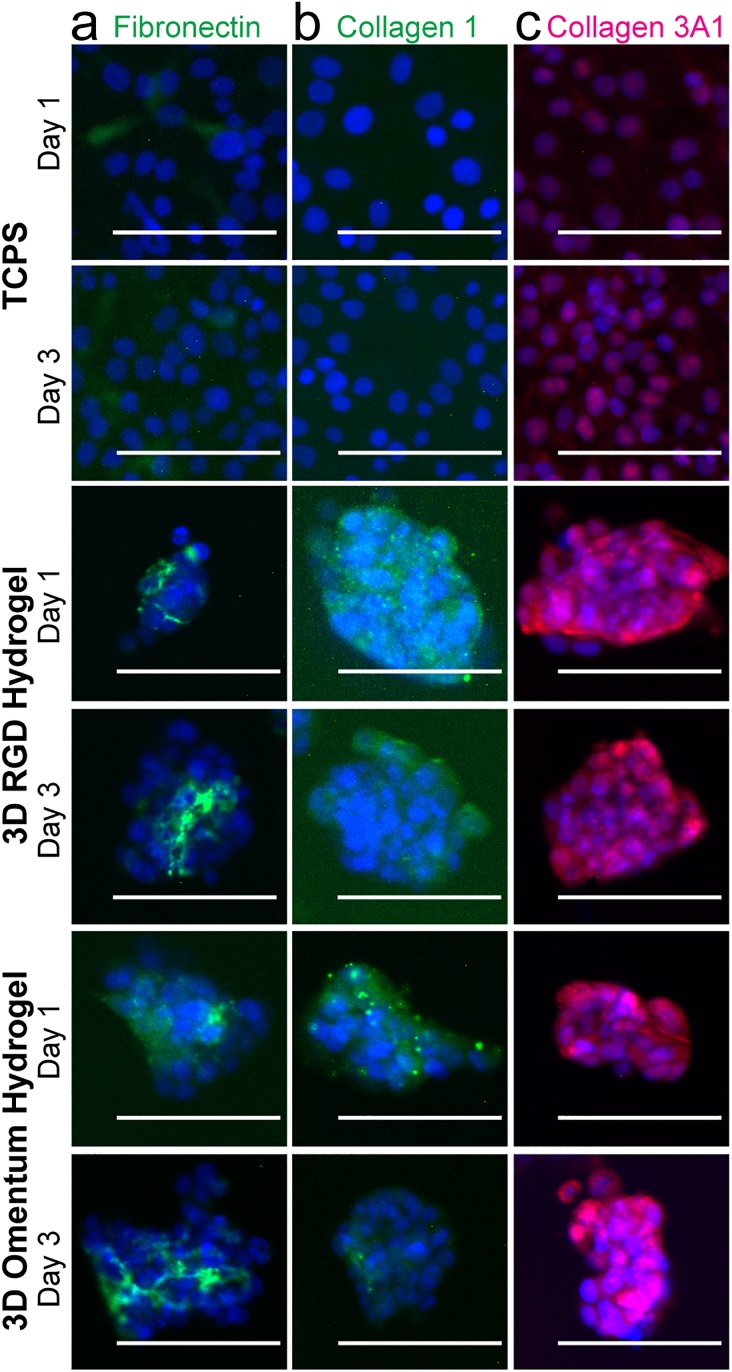
Immunofluorescence staining of SKOV-3 cells demonstrated that they produce their own ECM proteins over the time frame of a drug screening assay. (a) Fibronectin (green)/DAPI (blue). Fibronectin can be seen on plastic as well as between cells in the MCTS. (b) Collagen 1 (green)/DAPI (blue). Collagen 1 production was not observed on plastic but was visible around the spheroids. (c) Collagen3A1 (pink)/DAPI (blue). Some collagen 3A1 may be present on plastic but is clearly visible surrounding the spheroids in both hydrogels. Scale bars = 100 *μ*m. Images are representative from N ≥ 2 independent experiments.

To explore possible reasons for the differences in drug responses that we observed, we quantified the presence of a subset of ECM proteins via immunofluorescence staining (Fig. 4). Fibronectin has been shown to be produced by mesothelial cells to promote OvCa metastasis,^43^ and we observed fibronectin production on TCPS and in 3D hydrogels by the OvCa cells themselves [Fig. 4(a)]. By visual inspection, in both 3D hydrogel models, it appeared that the fibronectin was localized to within the MCTS rather than around the MCTS surface. Collagen 1 is a major component of the omentum microenvironment,^36^ and the work by Januchowski *et al.* discovered fibrillar collagen genes and, in particular, collagen 3A1 was upregulated in Topotecan- and Paclitaxel-resistant OvCa cells on TCPS.^44^ Therefore, we looked for both extracellular collagen 1 and collagen 3 (detected by collagen 1 and collagen 3A1 antibodies, respectively) in the environments tested here. We found that in the 3D hydrogels, but not on TCPS, SKOV-3 cells produced collagen 1 [Fig. 4(b)]. This suggests that perhaps the SKOV-3 MCTS were working to produce their own fibrillar collagen 1 for survival since GFOGER does not provide a fibrillar structure for the cells to most effectively bind. Furthermore, we observed some collagen 3A1 on TCPS on day 3, whereas it was highly expressed in both 3D conditions around and within the MCTS at both timepoints [Fig. 4(c)]. Since gene expression of this protein was upregulated for Paclitaxel resistant cell lines on TCPS,^44^ perhaps we are demonstrating that these MCTS produce collagen 3A1 to become Paclitaxel resistant in 3D. These results suggest that SKOV-3 cells may produce their own ECM proteins to increase their survival to prevent the response to Paclitaxel exposure in 3D. Future work could examine a larger panel of ECM proteins to attempt at correlating the presence on specific proteins and OvCa drug resistance in 3D microenvironments.

## DISCUSSION

There is clear room for improvement in OvCa treatment. The availability of ascites from OvCa patients provides a valuable opportunity for developing an improved OvCa drug testing pipeline that combines patient cells and engineered environments. Others have recognized the need for developing more clinically relevant OvCa drug screening platforms.^40,45^ Similar to our RGD hydrogel, Loessner *et al.* evaluated the response of SKOV-3 MCTS to Paclitaxel in a 3D PEG-based hydrogel with RGD binding sites and MMP-degradable sites.^40^ The MCTS generation approach was different from ours where their platform allowed for the formation of the MCTS from single cells within the PEG hydrogel over 12 days rather than by the aggregation and encapsulation method that we used.^40^ Even with a different 3D hydrogel and the MCTS formation method, they also observed resistance to Paclitaxel in 3D.^40^ However, they did not test the response to other drugs in their study, which limits what we can compare between studies. Liu *et al.*, with 3D collagen gels, observed resistance of OV-NC and OV-206 OvCa cell lines seeded as single cells in 3D to Paclitaxel, Carboplatin, and 5-fluorouracil relative to TCPS.^46^ Furthermore, the use of this collagen gel demonstrated that the ability of OvCa to interact with the collagen matrix increased the invasive capacity of the cells.^46^ From this observation and our collagen staining results, it appears that collagen 1 is necessary for OvCa disease progression and/or drug resistance in 3D and OvCa cells will produce this protein themselves if it is not provided within the biomaterial environment. To model the omentum for understanding OvCa metastasis, a multicellular platform containing mesothelial cells, fibroblasts, and ECM was developed.^47^ The application of this platform identified that mesothelial cells from the omentum caused inhibition and fibroblasts and ECM promoted attachment and invasion of OvCa cells.^47^ In the context of OvCa drug screening and the omentum microenvironment, we see our study as a valuable contribution to OvCa drug response research because we compare OCAS derived from ascites with cell line MCTS to aid in establishing links between preclinical drug screening models and clinical responses.

We suggest that our synthetic hydrogel model holds potential as a good tool for screening the patient OCAS drug response. By controlling the polymer concentration of the 3D hydrogels, we were able to test the samples encapsulated in hydrogels with a stiffness of 2.3 ± 1.9 kPa,^22^ which is physiologically relevant for omental tissue [2.9 ± 1.5 kPa (Ref. 34)]. Furthermore, the 3D PEG-MAL hydrogels are easy to make because all the peptides and polymer precursors can be made in advance and simply require dissolution for sample encapsulation. Thus, whenever patient samples are available, one can process the sample and complete the encapsulation within a few hours. Additionally, an ascites sample has limited OCAS material for screening, which makes our small volume hydrogel system useful for screening multiple compounds. If multiple treatment options are being considered for a patient, then this could be a simple, but valuable tool in guiding the selection of the next treatment steps. For example, LY2606368 is currently in Phase II clinical trials^41,42^ and could be an option for patients in the future. Testing this compound in 3D hydrogels demonstrated that it was more promising as a patient treatment than screening only on TCPS by comparing the reported EC_50_ values [Fig. 3(c)]. Furthermore, patient sample 1_2_ was received about 6 weeks after patient sample 1, and we observed an increase in resistance to the drugs that we tested. Our results imply that as disease progresses, the patient's ability to respond to treatment decreases for these particular samples. To our knowledge, Mafosfamide has never been tested in clinical trials for OvCa but has shown promise *in vitro* on TCPS.^48,49^ We could not find any additional information about further preclinical studies of this drug. We can only speculate that there may have been unpublished animal studies where Mafosfamide was not effective. We were interested in testing Mafosfamide as an example of a potential treatment for OvCa with our tissue-specific hydrogel system and found cells in the 3D hydrogel more sensitive to this drug compared to TCPS. The results from our 3D hydrogel approach suggest that this drug could be beneficial for patients, and this prediction of efficacy was likely not identified with earlier studies that relied solely on TCPS [Figs. 3(c) and S6]. If the additional integrin-binding peptides of the omentum hydrogel more faithfully capture the *in vivo* microenvironment, then this could potentially help explain how some compounds have been able to successfully become clinical OvCa treatments. However, it should be noted that the added complexity of additional integrin-binding peptides in the omentum hydrogel was not necessary for observing the SKOV-3 resistant to Paclitaxel in 3D. From our experiments, however, it is not clear if the 3D MCTS structure or if the 3D hydrogel environment is driving this lack of response across the range of concentrations of Paclitaxel what we tested. Overall, we suggest that our approach could and should be extrapolated to more possible OvCa drugs either originally overlooked or currently under consideration/development.

In our previously published work, we compared differences in gene expression of MCTS created using a variety of different methods (i.e., clonal growth in polyNIPAAM vs aggregation in microwells).^24^ In this first generation approach, we created MCTS by clonal growth from SKOV-3 cells and compared their response and the response of OCAS from patients with a small panel of drugs in a nondegradable, RGD integrin-binding, 3D synthetic PEG hydrogel.^24^ The results suggested that further exploration of OvCa in a 3D model may be useful for understanding the drug response in patient samples. Here, we used microwells, an aggregation formation method, to create MCTS since OCAS may form by aggregation in patients.^33^ By simply encapsulating the cells and OCAS from ascites, we preserved the cell-cell contacts and any associated ECM directly from the patient. Because we were limited in sample sizes, we are unsure of how much ECM comes with the OCAS, but we observed that the SKOV-3 cell line had the presence of secreted fibronectin, collagen 3, and collagen 1 within one day of encapsulation (Fig. 4). Since these ECM proteins were not seen on TCPS for all these cases, we suspect that ECM production facilitated survival in 3D by adding new integrin-binding sites in addition to those we provided via peptides. Our results aid in identifying collagen 3 as an important contributor to OvCa drug resistance since it was identified to be coexpressed with the ECM protein lumican in a Topotecan-resistant OvCa cell line on 2D by another research group.^50^ Perhaps this suggests that future work could potentially explore how these ECM proteins directly impact the OvCa response to drug.

It has been well-established that cells grow at different rates in 2D and 3D cultures.^13,51^ Since the ability of cells to respond to chemotherapy is proliferation-dependent, the response is expected to vary depending on growth conditions. To account for differences in growth rates that will impact the measurements for traditional drug screening metrics, the GR_50_ was established.^39^ One key condition that must be met when applying this analysis is that the cells are growing exponentially over the course of the assay.^39,52^ We have found that this key condition is not always met, especially in biomaterial platforms, and has discussed this in further detail.^38^ In this study, we were not able to calculate the GR_50_ for all the conditions that we tested due to growth limitations. Therefore, the EC_50_ is what we were able to report here for all the conditions that we tested. Due to the differences in growth rates of cells in 2D and 3D, our 3D model helps aid in understanding growth of the MCTS in 3D *in vitro* before moving to *in vivo* studies. We tested drugs used clinically to treat patients, a drug that is in clinical trials, and Mafosfamide, which has shown promise *in vitro* on TCPS,^48,49^ but to our knowledge, has never been studied in clinical trials. As more people come to know about how the microenvironment impacts the drug response, it may be possible to evaluate additional quantitative phenotypes that would aid in comparing drug responses across different geometries. Others have created models with other cell types including mesothelial cells and fibroblasts,^47,53,54^ but here our system was focused on the parameters of integrin-binding sites, stiffness, and 3D geometry without the contribution of other cells present within the omentum microenvironment. Since ascites is rich in cytokines and growth factors,^32^ perhaps future studies with patient-derived OCAS could incorporate ascites-derived cytokines as an additional feature for more faithfully modeling the omentum microenvironment for OvCa drug screening. By evaluating the presence of a subset of ECM proteins in addition to the EC_50_ for each condition, we have enabled an approach to couple the microenvironment composition and drug response, which could be applied in other drug screening studies with biomaterials. This could help establish large drug response and ECM composition data sets, which could then possibly be used to determine correlations between the ECM composition and the drug response that may aid in making better clinical predictions for the success of a drug candidate.

OvCa is a challenging disease to treat and the development of second line therapies is critical since recurrence occurs in a majority of patients. We propose that this can be achieved by using a 3D PEG-MAL hydrogel with encapsulated MCTS to aid in making better preclinical predictions for new therapeutic compounds. Our results demonstrated that screening for therapies on TCPS and in 3D hydrogels did not yield the same drug responses. This observation may be dependent on the ECM composition, and we concluded that TCPS may not be sufficient for evaluation of the OvCa drug response. Most patients have the presence of ascites, which is removed as part of treatment and contains many OCAS. The integration of OCAS in an omentum-inspired 3D hydrogel model to study the drug response yielded results that are also different from those of TCPS. We discovered that our 3D hydrogel model demonstrated drug response results that we could not have captured on TCPS alone and recommend that future OvCa drug screens employ our materials to aid in understanding the OvCa response *in vitro*.

## METHODS

### Cell culture

All cells were maintained at 37 °C and 5% CO_2_, and all cell culture supplies were purchased from Thermo Fisher Scientific (Waltham, MA) unless otherwise noted. The SKOV-3 cell line was purchased from the American Type Culture Collection (ATCC, Manassas, VA). SKOV-3 cells were grown in Roswell Park Memorial Institute (RPMI) medium supplemented with 10% fetal bovine serum (FBS) and 1% penicillin/streptomycin (pen/strep).

### Primary ovarian cancer ascites culture

Ascites samples were received from patients undergoing paracentesis at UMass Medical School (Worcester, MA), transported to UMass Amherst the same day as the procedure and used for experiments immediately upon receipt. Samples were deidentified and were IRB exempt. Pathology reports are provided in Fig. 3(a). Single cells and OCAS were recovered from patient samples and treated as described previously.^24^ Samples containing both single cells and OCAS were grown on 2D TCPS or encapsulated directly into 3D PEG-MAL hydrogels. All experiments with primary OvCa samples were conducted as one biological replicate with RPMI + 10% FBS + 1% pen/strep.

### Microwell MCTS

Square pyramidal microwells (400 *μ*m sidewall dimension) were fabricated as described previously.^55^ Briefly, master molds containing square-pyramidal pits were generated by anisotropic etching of 100 crystalline silicon in potassium hydroxide (KOH). Microwells were generated from poly(dimethylsiloxane) (PDMS) using a two-stage replica molding process of the master mold as described previously.^55^ Microwells were arranged in a square array with no space between adjacent wells and placed in 12-well plates. For cell seeding, microwell surfaces were UV sterilized for 30 min, pretreated with 5% Pluronic F-127 (Sigma-Aldrich, St. Louis, MO) for 5 min at 3500 RPM (2465×g, Eppendorf 5810R v3.3 centrifuge with A-4-62 rotor, Eppendorf, Hamburg, Germany) with an additional 30-min incubation on the bench, and then washed twice with sterile water. Cells were seeded 1.00 × 10^5^ cells/well (approximately 26 000 cells/cm^2^). After 24 h, MCTS were collected by shaking the plate gently to dislodge most of them and gently aspirating medium and MCTS. MCTS solution was spun down at 400 RPM (Thermo Scientific Sorvall ST16R centrifuge) for 5 min. Medium was removed, MCTS were encapsulated in 3D hydrogels, and RPMI + 5% FBS + 1% pen/strep was added to each well after the hydrogels had polymerized.

### 3D PEG-MAL hydrogel platform

MCTS or OCAS were encapsulated into 3D PEG hydrogels as described previously^24^ with minor modifications. The 3D RGD hydrogel was prepared with a 20 kDa 4-arm PEG-maleimide (PEG-MAL, Jenkem Technology, Plano, TX) at 10 wt. % solution [measured Young's modulus at this condition was 2.3 ± 1.9 kPa (Ref. 22) and corresponds to the initial average measured elastic modulus of omental tissue of 2.9 ± 1.5 kPa (Ref. 34)]. The RGD hydrogels also included 2 mM of cell adhesion peptide GSPCRGDG (RGD, Genscript, Piscataway, NJ) and cross-linked with a 90 mM combination of the 1 kDa linear PEG-dithiol (JenKem, 79.2 mM) and cell-degradable cross-linker, Pan-matrix metalloproteinase (Pan-MMP, GCRDGPQGIWGQDRCG, Genscript, 10.8 mM)^35^ in sterile 2 mM triethanolamine (pH 7.4). The hydrogels were synthesized by pipetting 1 *μ*l of PDT and Pan-MMP solution onto the well surface of a 48-well TCPS plate, followed by pipetting 9 *μ*l of the MCTS/OCAS-containing PEG—MAL solution. The 3D omentum hydrogel was prepared with 2 mM of the omentum integrin-binding peptide cocktail. The omentum cocktail is fully described in Fig. 1(c) and consists of (GRGDSPCG, Genscript), GFOGER (CGP(GPP)_5_GFOGER(GPP)_5_, Genscript), PSHRN-RGD (CGPHSRNGGGGGGRGDS, the same methods as described previously^22^), DGEA (GCGDGEA, the same methods as described previously^22^). The MCTS from microwells were transferred to either a 3D RGD hydrogel or 3D omentum hydrogel at a ratio of one well of a 12-well microwell plate to six 10 *μ*l PEG-MAL hydrogels. The PEG-MAL solution containing MCTS was transferred with cut pipette tips to minimize shear stress.

### Drug screening

Single SKOV-3 cells were seeded in RPMI with 5% FBS and 1% pen/strep at 6250 cell/cm^2^ on TCPS 96-well plates. SKOV-3 MCTS were recovered and encapsulated in 3D hydrogels with the same medium as TCPS. Samples collected from ascites were seeded in RPMI with 10% FBS and 1% pen/strep at the same density on TCPS. Drugs were added after 24 h in the same type of medium that the cells were seeded in and the cells were incubated with drugs for 48 h. Carboplatin (Tocris Bioscience, United Kingdom) was added in tenfold serial dilutions at concentrations of 2 × 10^−5^ to 2 × 10^2^ *μ*M. Paclitaxel (MP Biomedicals, Santa Ana, CA), Mafosfamide (Niomech, Germany), and Doxorubicin (LC Laboratories, Woburn, MA) were added in tenfold serial dilutions at concentrations of 1 × 10^−5^ to 1 × 10^2^ *μ*M. LY2606368 (AbovChem LLC, San Diego, CA) was added in tenfold serial dilutions at concentrations of 3 × 10^−5^ to 3 × 10^1^ *μ*M. Dimethyl sulfoxide (DMSO, Sigma-Aldrich) was used as a vehicle control for all drugs except Carboplatin was dissolved in water.^56^ Cell viability was assayed at 24 h postcell seeding in a control plate and after 48 h of drug incubation using the CellTiter-Glo luminescent viability assay (Promega, Madison, WI). After five (TCPS) or 20 (hydrogels) min after adding the CellTiter-Glo, luminescence values were measured using a BioTek Synergy H1 plate reader (BioTek, Winooski, VT). The drug response metrics were calculated using the online GR calculator^39^ found at http://www.grcalculator.org/grcalculator/ for each cell line and drug combination. For the input file format, we used the initial cell count calculation method and Case A (multiple cell counts per row). The variable “replicate” was removed from the grouping variable list before the data were analyzed. However, the EC_50_ values for LY2606368 drug responses of patient 1 and the second biological replicate of SKOV-3 MCTS in the omentum-inspired hydrogel were calculated using GraphPad Prism v7.0c (GraphPad Software, Inc., LaJolla, CA) due to limitations of the GR calculator with the drug response curves in these cases. Conditions that were reported as “did not respond in 3D” were determined by meeting one of or both the following criteria: (1) the output of the GR calculator defined the relative cell count fit (rather than the GR fit) as “flat” rather than “sigmoid.” Or (2) all the plotted data points ± the standard error of the mean fit within the average drug response ± the standard deviation across all concentrations that were tested. The SKOV-3 data reported are from N ≥ 2 independent experiments with N ≥ 2 technical replicates in each experiment.

### Immunofluorescence (IF) staining

All antibodies and corresponding dilutions used for IF staining are listed in Table S1. Single cells seeded on TCPS or MCTS encapsulated in 3D hydrogels for 1 or 3 days were assessed for proliferation via Ki67 immunofluorescence. For the Ki67 staining, samples were rinsed with phosphate buffered saline (PBS), fixed with 10% formalin for 15 min, permeabilized with tris-buffered saline (TBS) containing 0.5% Triton X-100 (Promega), and blocked with AbDil [2 wt. % bovine serum albumin (BSA, Thermo) in TBS with 0.1% Triton X-100, TBS-T] for 20 min. Samples were incubated for 1 (TCPS) or 2 (hydrogels) hours at room temperature with the primary antibody, rinsed three times with TBS-T, and incubated with a secondary antibody for 1 (TCPS) or 2 (hydrogels) hours. To stain for ECM proteins, the cells were incubated with the antibodies listed in Table S1 for the incubation times described above. Cell nuclei were labeled with DAPI (Thermo) at 1:1000 in phosphate buffered saline (PBS) for five min, and samples were rinsed three times with PBS. Fluorescence imaging was performed on a Zeiss Spinning Disc Cell Observer SD (Carl Zeiss AG, Oberkochen, Germany). The brightness and contrast were adjusted post-image collection, and SKOV-3 images are representative from N ≥ 2 independent experiments.

### LIVE/DEAD cell viability staining

Cells seeded on TCPS or MCTS encapsulated in 3D hydrogels for 1 and 3 days were assessed for viability with LIVE/DEAD staining (L3224, Thermo) in accordance with the manufacturer's instructions. Imaging was obtained on a Zeiss Spinning Disc Cell Observer SD (Zeiss). The brightness and contrast were adjusted postimage collection, and SKOV-3 images are representatives from N ≥ 2 independent experiments.

### Statistical analysis

Statistical analysis was performed using GraphPad Prism v7.0c (GraphPad). Statistical significance was evaluated using a one-way analysis of variance (ANOVA) followed by a Tukey's posttest for pairwise comparisons. For the analysis, p-values <0.05 are considered significant, where p < 0.05 is denoted with ^*^, ≤0.01 with ^**^, ≤0.001 with ^***^, and ≤0.0001 with ^****^.

## SUPPLEMENTARY MATERIAL

See the supplementary material for antibodies used for immunofluorescence staining, data on hydrogel optimization, and complete drug response curves.

## References

[1] R. L. Siegel , K. D. Miller , and A. Jemal , CA: Cancer J. Clin. 68(1), 7–30 (2018). 10.3322/caac.21442 29313949

[2] R. F. Ozols , M. A. Bookman , D. C. Connolly , M. B. Daly , A. K. Godwin , R. J. Schilder , X. Xu , and T. C. Hamilton , Cancer Cell 5, 19–24 (2004). 10.1016/S1535-6108(04)00002-9 14749123

[3] D. S. P. Tan , R. Agarwal , and S. B. Kaye , Lancet Oncol. 7, 925–934 (2006). 10.1016/S1470-2045(06)70939-1 17081918

[4] E. Lengyel , Am. J. Pathol. 177(3), 1053–1064 (2010). 10.2353/ajpath.2010.100105 20651229 PMC2928939

[5] M.-L. Puiffe , C. Le Page , A. Filali-Mouhim , M. Zietarska , V. Ouellet , P. N. Tonin , M. Chevrette , D. M. Provencher , and A.-M. Mes-Masson , Neoplasia 9(10), 820–829 (2007). 10.1593/neo.07472 17971902 PMC2040209

[6] S. Al Habyan , C. Kalos , J. Szymborski , and L. McCaffrey , Oncogene 37, 5127–5135 (2018). 10.1038/s41388-018-0317-x 29789717 PMC6137025

[7] S. Vaughan , J. I. Coward , R. C. Bast, Jr. , A. Berchuck , J. S. Berek , J. D. Brenton , G. Coukos , C. C. Crum , R. Drapkin , D. Etemadmoghadam , M. Friedlander , H. Gabra , S. B. Kaye , C. J. Lord , E. Lengyel , D. A. Levine , I. A. McNeish , U. Menon , G. B. Mills , K. P. Nephew , A. M. Oza , A. K. Sood , E. A. Stronach , H. Walczak , D. D. Bowtell , and F. R. Balkwill , Nat. Rev. Cancer 11, 719–719 (2011). 10.1038/nrc3144 21941283 PMC3380637

[8] R. Agarwal and S. B. Kaye , Nat. Rev. Cancer 3(7), 502–516 (2003). 10.1038/nrc1123 12835670

[9] K. Pliarchopoulou and D. Pectasides , Crit. Rev. Oncol./Hematol. 79(1), 17–23 (2011). 10.1016/j.critrevonc.2010.07.004 20674385

[10] P. Horvath , N. Aulner , M. Bickle , A. M. Davies , E. D. Nery , D. Ebner , M. C. Montoya , P. Östling , V. Pietiäinen , L. S. Price , S. L. Shorte , G. Turcatti , C. V. Schantz , and N. O. Carragher , Nat. Rev. Drug Discovery 15(11), 751–769 (2016). 10.1038/nrd.2016.175 27616293

[11] S. A. Langhans , Front. Pharmacol. 9, 1–14 (2018). 10.3389/fphar.2018.00006 29410625 PMC5787088

[12] R. Edmondson , J. J. Broglie , A. F. Adcock , and L. Yang , Assay Drug Dev. Technol. 12(4), 207–218 (2014). 10.1089/adt.2014.573 24831787 PMC4026212

[13] B. M. Baker and C. S. Chen , J. Cell Sci. 125(13), 3015–3024 (2012). 10.1242/jcs.079509 22797912 PMC3434846

[14] K. S. M. Smalley , M. Lioni , and M. Herlyn , In Vitro Cell. Dev. Biol.-Anim. 42, 242–247 (2006). 10.1290/0604027.1 17163781

[15] K. L. Schmeichel and M. J. Bissell , J. Cell Sci. 116(12), 2377–2388 (2003). 10.1242/jcs.00503 12766184 PMC2933213

[16] M. W. Tibbitt and K. S. Anseth , Biotechnol. Bioeng. 103(4), 655–663 (2009). 10.1002/bit.22361 19472329 PMC2997742

[17] L. Jansen , T. McCarthy , M. Lee , and S. Peyton , preprint bioRxiv (2018).

[18] E. A. Phelps , N. O. Enemchukwu , V. F. Fiore , J. C. Sy , N. Murthy , T. A. Sulchek , T. H. Barker , and A. J. Garcia , Adv. Mater. 24(1), 64–70 (2012). 10.1002/adma.201103574 22174081 PMC3517145

[19] B. V. Sridhar , J. L. Brock , J. S. Silver , J. L. Leight , M. A. Randolph , and K. S. Anseth , Adv. Healthcare Mater. 4(5), 702–713 (2015). 10.1002/adhm.201400695 PMC4487633 25607633

[20] K. M. Mabry , M. E. Schroeder , S. Z. Payne , and K. S. Anseth , ACS Appl. Mater. Interfaces 8(34), 21914–21922 (2016). 10.1021/acsami.5b11359 27050338

[21] S. Pedron , E. Becka , and B. A. Harley , Adv. Mater. 27(9), 1567–1572 (2015). 10.1002/adma.201404896 25521283

[22] E. A. Brooks , L. E. Jansen , M. F. Gencoglu , A. M. Yurkevicz , and S. R. Peyton , ACS Biomater. Sci. Eng. 4(2), 707–718 (2018). 10.1021/acsbiomaterials.7b00737 33418758

[23] M. Caiazzo , Y. Okawa , A. Ranga , A. Piersigilli , Y. Tabata , and M. P. Lutolf , Nat. Mater. 15, 344–352 (2016). 10.1038/nmat4536 26752655

[24] M. F. Gencoglu , L. E. Barney , C. L. Hall , E. A. Brooks , A. D. Schwartz , D. C. Corbett , K. R. Stevens , and S. R. Peyton , ACS Biomater. Sci. Eng. 4(2), 410–420 (2018). 10.1021/acsbiomaterials.7b00069 29527571 PMC5843470

[25] S. Domcke , R. Sinha , D. A. Levine , C. Sander , and N. Schultz , Nat. Commun. 4(2126), 1–10 (2013). 10.1038/ncomms3126 PMC3715866 23839242

[26] J.-P. Gillet , S. Varma , and M. M. Gottesman , J. Natl. Cancer Inst. 105(7), 452–458 (2013). 10.1093/jnci/djt007 23434901 PMC3691946

[27] A. Goodspeed , L. M. Heiser , J. W. Gray , and J. C. Costello , Mol. Cancer Res. 14(1), 3–13 (2016). 10.1158/1541-7786.MCR-15-0189 26248648 PMC4828339

[28] T. Ishiguro , H. Ohata , A. Sato , K. Yamawaki , T. Enomoto , and K. Okamoto , Cancer Sci. 108(3), 283–289 (2017). 10.1111/cas.13155 28064442 PMC5378268

[29] A. M. Patch , E. L. Christie , D. Etemadmoghadam , D. W. Garsed , J. George , S. Fereday , K. Nones , P. Cowin , K. Alsop , P. J. Bailey , K. S. Kassahn , F. Newell , M. C. J. Quinn , S. Kazakoff , K. Quek , C. Wilhelm-Benartzi , E. Curry , H. S. Leong , A. Hamilton , L. Mileshkin , G. Au-Yeung , C. Kennedy , J. Hung , Y. E. Chiew , P. Harnett , M. Friedlander , M. Quinn , J. Pyman , S. Cordner , P. O'Brien , J. Leditschke , G. Young , K. Strachan , P. Waring , W. Azar , C. Mitchell , N. Traficante , J. Hendley , H. Thorne , M. Shackleton , D. K. Miller , G. M. Arnau , R. W. Tothill , T. P. Holloway , T. Semple , I. Harliwong , C. Nourse , E. Nourbakhsh , S. Manning , S. Idrisoglu , T. J. C. Bruxner , A. N. Christ , B. Poudel , O. Holmes , M. Anderson , C. Leonard , A. Lonie , N. Hall , S. Wood , D. F. Taylor , Q. Xu , J. L. Fink , N. Waddell , R. Drapkin , E. Stronach , H. Gabra , R. Brown , A. Jewell , S. H. Nagaraj , E. Markham , P. J. Wilson , J. Ellul , O. McNally , M. A. Doyle , R. Vedururu , C. Stewart , E. Lengyel , J. V. Pearson , N. Waddell , A. Defazio , S. M. Grimmond , and D. D. L. Bowtell , Nature 521(7553), 489–494 (2015). 10.1038/nature14410 26017449

[30] A. Letai , Nat. Med. 23(9), 1028–1035 (2017). 10.1038/nm.4389 28886003

[31] E. Kipps , D. S. P. Tan , and S. B. Kaye , Nat. Rev. Cancer 13(4), 273–282 (2013). 10.1038/nrc3432 23426401 PMC4673904

[32] N. Ahmed and K. L. Stenvers , Front. Oncol. 3, 1–12 (2013). 10.3389/fonc.2013.00256 24093089 PMC3782691

[33] K. Shield , M. L. Ackland , N. Ahmed , and G. E. Rice , Gynecol. Oncol. 113(1), 143–148 (2009). 10.1016/j.ygyno.2008.11.032 19135710

[34] N. Alkhouli , J. Mansfield , E. Green , J. Bell , B. Knight , N. Liversedge , J. C. Tham , R. Welbourn , A. C. Shore , K. Kos , and C. P. Winlove , Am. J. Physiol.-Endocrinol. Metab. 305(12), E1427–E1435 (2013). 10.1152/ajpendo.00111.2013 24105412

[35] M. P. Lutolf , J. L. Lauer-Fields , H. G. Schmoekel , A. T. Metters , F. E. Weber , G. B. Fields , and J. A. Hubbell , PNAS 100(9), 5413–5418 (2003). 10.1073/pnas.0737381100 12686696 PMC154359

[36] A. Naba , O. M. T. Pearce , A. Del Rosario , D. Ma , H. Ding , V. Rajeeve , P. R. Cutillas , F. R. Balkwill , and R. O. Hynes , J. Proteome Res. 16(8), 3083–3091 (2017). 10.1021/acs.jproteome.7b00191 28675934 PMC8078728

[37] W.-M. Zhang , J. Käpylä , J. S. Puranen , C. G. Knight , C.-F. Tiger , O. T. Pentikäinen , M. S. Johnson , R. W. Farndale , J. Heino , and D. Gullberg , J. Biol. Chem. 278(9), 7270–7277 (2003). 10.1074/jbc.M210313200 12496264

[38] E. A. Brooks , S. Galarza , M. F. Gencoglu , R. C. Cornelison , J. M. Munson , and S. Peyton , preprint bioRxiv (2018).

[39] M. Hafner , M. Niepel , M. Chung , and P. K. Sorger , Nat. Methods 13(6), 521–527 (2016). 10.1038/nmeth.3853 27135972 PMC4887336

[40] D. Loessner , K. S. Stok , M. P. Lutolf , D. W. Hutmacher , J. A. Clements , and S. C. Rizzi , Biomaterials 31(32), 8494–8506 (2010). 10.1016/j.biomaterials.2010.07.064 20709389

[41] J.-M. Lee , N. Jayakumar , A. Zimmer , S. Lipkowitz , C. M. Annunziata , M. J. Merino , E. M. Swisher , M. I. Harrell , J. B. Trepel , M.-J. Lee , M. H. Bagheri , D.-A. Botesteanu , S. M. Steinber , L. Minasian , I. Ekwede , and E. C. Kohn , Lancet Oncol. 19(2), 207–215 (2018). 10.1016/S1470-2045(18)30009-3 29361470 PMC7366122

[42] J.-M. Lee , F. H. Karzai , A. Zimmer , C. M. Annunziata , S. Lipkowitz , B. Parker , N. Houston , I. Ekwede , and E. C. Kohn , Ann. Oncol. 27, vi296–vi312 (2016). 10.1093/annonc/mdw374.02

[43] H. A. Kenny , C. Y. Chiang , E. A. White , E. M. Schryver , M. Habis , I. L. Romero , A. Ladanyi , C. V. Penicka , J. George , K. Matlin , A. Montag , K. Wroblewski , S. D. Yamada , A. P. Mazar , D. Bowtell , and E. Lengyel , J. Clin. Invest. 124(10), 4614–4628 (2014). 10.1172/JCI74778 25202979 PMC4191043

[44] R. Januchowski , M. Świerczewska , K. Sterzyńska , K. Wojtowicz , M. Nowicki , and M. Zabel , J. Cancer 7(10), 1295–1310 (2016). 10.7150/jca.15371 27390605 PMC4934038

[45] S. Raghavan , M. R. Ward , K. R. Rowley , R. M. Wold , S. Takayama , R. J. Buckanovich , and G. Mehta , Gynecol. Oncol. 138(1), 181–189 (2015). 10.1016/j.ygyno.2015.04.014 25913133 PMC4480341

[46] M. Liu , X. Zhang , C. Long , H. Xu , X. Cheng , J. Chang , C. Zhang , C. Zhang , and X. Wang , RSC Adv. 8(16), 8910–8919 (2018). 10.1039/C7RA13742G PMC9078576 35539845

[47] H. A. Kenny , T. Krausz , S. D. Yamada , and E. Lengyel , Int. J. Cancer 121(7), 1463–1472 (2007). 10.1002/ijc.22874 17546601

[48] D. S. Alberts , J. G. Einspahr , R. Struck , G. Bignami , L. Young , E. A. Surwit , and S. E. Salmon , Invest. New Drugs 2(2), 141–148 (1984). 10.1007/BF00232343 6469507

[49] A. O. Mueck , H. Seeger , D. Wallwiener , and J. Huober , Eur. J. Gynaecol. Oncol. 25(6), 699–701 (2004). 15597845

[50] A. Klejewski , K. Sterzynska , K. Wojtowicz , M. Swierczewska , M. Partyka , M. Brazert , M. Nowicki , M. Zabel , and R. Januschowski , Oncotarget 8(43), 74466–74478 (2017). 10.18632/oncotarget.20169 29088800 PMC5650355

[51] M. Ravi , A. Ramesh , and A. Pattabhi , J. Cell. Physiol. 232(10), 2679–2697 (2017). 10.1002/jcp.25664 27791270

[52] M. Hafner , L. M. Heiser , E. H. Williams , M. Niepel , N. J. Wang , J. E. Korkola , J. W. Gray , and P. K. Sorger , Sci. Data 4, 170166 (2017). 10.1038/sdata.2017.166 29112189 PMC5674849

[53] F. Xu , J. Celli , I. Rizvi , S. Moon , T. Hasan , and U. Demirci , Biotechnol. J. 6(2), 204–212 (2011). 10.1002/biot.201000340 21298805 PMC3780785

[54] M. Lal-Nag , L. McGee , R. Guha , E. Lengyel , H. A. Kenny , and M. Ferrer , SLAS Discovery 22(5), 494–506 (2017). 10.1177/2472555216687082 28346091 PMC5815521

[55] K. R. Stevens , M. D. Ungrin , R. E. Schwartz , S. Ng , B. Carvalho , K. S. Christine , R. R. Chaturvedi , C. Y. Li , P. W. Zandstra , C. S. Chen , and S. N. Bhatia , Nat. Commun. 4, 1847 (2013). 10.1038/ncomms2853 23673632 PMC3660041

[56] M. D. Hall , K. A. Telma , K.-E. Chang , T. D. Lee , J. P. Madigan , J. R. Lloyd , I. S. Goldlust , J. D. Hoeschele , and M. M. Gottesman , Cancer Res. 17(14), 3913–3923 (2014). 10.1158/0008-5472.CAN-14-0247 PMC4153432 24812268

